# Monthly Memoranda

**Published:** 1854-03

**Authors:** 


### EDITORIAL DEPARTMENT.

### MONTHLY MEMORANDA.

*Alum, an emetic in Croup.*—In our Dec. No. we gave a case in which alum was favorably employed by Dr. Meigs, as an emetic, in a serious instance of poisoning by opium. A writer in the N. H. Journal of Medicine alludes to its uses in *true* croup. The authority of an aged and respectable physician of S. Carolina, is quoted, who says that "for many years he had made use in his practice of alum emetics for breaking up the recent formations of false membrane in cases of true croup, and that he considered the practice not only entirely safe, but very successful."

The writer adds: "For three years I have used this remedy in croup, and in that time have not seen a fatal case which was treated with it from the beginning. I do not recommend it as a specific, but I believe it to be a very safe and efficient remedy. I have usually given about ten grs. once in ten minutes, until vomiting is induced. I do not omit, whilst using the alum, to use the tartar emetic or the hive syrup freely,—for this acts to subdue the inflammation, while the alum has more of a revulsive action."

*Spermatorrhœa.*—Every physician knows well the obstinacy of this disease. Whatever the mode of treatment employed, he often feels the need of some appliance which he may rely upon, as having a directly favorable influence upon the diseased and irritable condition. We cannot do our medical readers and their patients a better service than by calling attention to the excellent mechanical remedy lately invented by Dr. Cheever of Boston. In many cases of this obstinate disease, treatment by medicinal agents alone, will be quite unavailing, and speedy and permanent cure can only be hoped for from the use of some appliance like the one alluded to, which by a happy process, *keeps down* and permanently checks the unnatural irritability. Dr. Buck of Hartford has also invented a similar contrivance. We have obtained by letter, from Boston, the simple apparatus of Dr. Cheever, and have found its employment eminently successful. It can be easily forwarded by mail, to any physician ordering it, and we think its use will not disappoint those who may employ it.

*Fish diet.*—Dr. John Davy has published some interesting observations with regard to fish as a diet. He compares the varieties of fish with other kinds of animal food, as to their specific gravities, and their per cent. of solid matter. He concludes that salmon and mackerel are the most nutritive of fishes, and compare favorably with beef, pork and mutton. From his investigations, fish is entitled to hold a higher rank, among articles of a nutritive nature, than the old and popular notions have granted it. But the most interesting part of Dr. Davy's researches refers to the peculiar *qualities* of fish, as an article of diet—especially used as a preventative of tubercular disease. In a public hospital at Plymouth, of 654 cases of phthisis, only *four* were fishermen; but among the troops, who eat meat daily, but rarely taste fish, of 1205 cases of disease, 734 were tuberculous, or 61 per cent. The author infers that some element enters into the composition of fish, rendering it a useful agent in mitigating, if not preventing consumption. Perhaps it may be the same element that renders cod liver oil so efficacious in scrofulous diseases.

The Virginia Med. and Surg. Journal, from which we condense these facts states that "the author closes his paper with the remarkable statistical fact, that among 763 fishermen and pilots, and 608 shoemakers and cordwainers in Plymouth, it is found upon consulting the dispensary returns, that the total number of shoemakers who have died with tubercular disease has been 37; whereas, the total number of deaths from the same disease amongst the fishermen, the more numerous of the two, was, as we have seen, only four."

*Menorrhagia.*—Dr. Tanner states, in the London Lancet, of Dec. 1853, "that in those cases of increased catamenial flow unconnected with physical alterations, plethora, or a watery state of the blood, in which rest and the usual astringents are unavailing, he has derived great advantage from the tincture of cinnamon. It is well known, Dr. Pereira regarded cinnamon as having a direct action upon the uterus, and Dr. Tanner is convinced that its utility in these cases depends upon this special property, and not upon its general specific action.—The medicine is given in drachm doses, cinnamon-water being the vehicle. Its use should be continued for fourteen days after the disappearance of the symptoms."

In our last number we published an article describing a new and prompt method of producing emesis in cases of poisoning and the suggestion seems to be in general favor, judging from the attention which it has received at the hands of the journalists.

Three years since we were called upon in a case of constipation of the bowels, which defied all the ordinary remedial measures. After using the flexible rectum tube repeatedly, though unavailingly, it occurred to us that if the bowels could be distended with gas below, as they manifestly were above, the constriction, that it might be overcome and accordingly tartaric acid, to the amount of ʒij, was dissolved in water, to which thin starch was added with the view to protect the mucous lining of the bowels and also to furnish a more consistent fluid. This was thrown up the bowels through the flexible tube. Immediately after, the same amount of sup. carb. soda was dissolved in water and thrown up also with considerable force. The patient immediately complained of pain similar to colic and the ear applied to the abdomen detected an unusual rumbling noise. The patient loudly importuned for permission to go to the close stool, which, after a short time, was permitted, when the bowels were freely evacuated and recovery followed in a few days.

In March 1850, we were applied to by an elderly man, a laborer, with inquinal hernia, which caused him pain and much uneasiness. Prof. Armor was in the office at the time and saw the case. The patient was advised to retire to his boarding house where the writer found him in a short time. His bowels had been constipated for some days, which, together with the tenderness produced by pressure upon the tumor, and the excitement of the pulse, led us to fear strangulation. The ordinary efforts for its reduction were used but without success, previous to which attempts, a brisk carthartic had been given. After a reasonable delay, during which the bowels were not moved, we resorted to the rectum tube through which we threw up—first tepid water, then a solution of common salt, ol. ricini. turpentine and starch, ol. croton suspended in starch, the decoct. senna, &c., &c., and yet the bowels remained unmoved.

At this stage of the proceeding, it occurred to us that we had seen somewhere a hint as to the value of producing a vacuum in similar cases. We immediately acted upon the suggestion, and, after a few strokes of the piston, reversing the action a vacuum was produced, when the patient declared that he distinctly felt the tumor suddenly subside. Upon examination we were gratified to find that this was the case.

Although this is not an analagous case to the former, yet it shows the necessity which often arises of throwing ourselves upon our own resources. Had an operation been resorted to and resulted happily, it would have been regarded as a great triumph by a wondering populous; and yet because both the torture of the operation and its dangerous consequences were avoided by a proceeding, in itself both simple and painless, it will scarcely excite a thought in the mind. We are led into these remarks by reflecting upon this common evidence of popular injustice in the bestowment of extravagant encomiums upon surgical triumphs, when as much, yea even more, science has been, and is every day being displayed at the bed-side without the aid of the glittering displays of surgical appliances. We are proud of the triumphs of surgery and the profession can justly boast of its capacity for the performance of great achievements; but to say that it constitutes the chief feature in medical science, we positively deny. Humanity could as well and perhaps better spare that department than the others, and yet *with* the others it makes up a great system, abounding in resources and elements of great and substantial good to the human family.

Obstetric Operation.—Whilst engaged in the prosecution of our enquiries into the symptoms of a case a short time since, which proved to be a case of uterine phlebitis, occurring after a second parturition, a fact was elicited, which, for the boldnes of the measure and the individual who performed it, somewhat startled us. It appeared that in the patient's first labor, the head descended upon the perinæum, where it lay for a short time, and the midwife who was in attendance becoming discouraged, took a *razor* (*!*) and slit down the perinæum for a distance. She did not adopt the oblique incision by Dubois and others, of a line or two in length, but slashed down the median line an inch or more, ignorant of the probable consequences. The wound cicatrized and left a hard, unyielding band, more or less preventing the ready dilatation of the soft parts in a line, extending from the fourchette toward anus. It did not appear, however, that it constituted any serious impediment to the second parturition.

